# LRD-DETR: A Lightweight RT-DETR-Based Model for Road Distress Detection

**DOI:** 10.3390/s26082375

**Published:** 2026-04-12

**Authors:** Chen Dong, Yunwei Zhang

**Affiliations:** 1 Faculty of Information Engineering and Automation, Kunming University of Science and Technology, Kunming 650500, China; 13577395255@163.com; 2 Higher Educational Key Laboratory for Industrial Intelligence and Systems of Yunnan Province, Kunming 650500, China

**Keywords:** LRD-DETR, lightweight backbone network, distress regions, background interference

## Abstract

Intelligent road distress detection technology has emerged as an important research topic in the field of highway maintenance. However, the accuracy and practicality of pavement distress detection are constrained by multiple factors, primarily including the irregular shapes of distress, the tendency for fine cracks to be overlooked, and the high parameter count of detection models that makes deployment difficult. Therefore, this study proposes a lightweight road distress detection model based on an improved RT-DETR architecture—LRD-DETR. First, this work integrates the C2f-LFEM module with the ADown adaptive down-sampling strategy into the backbone network, significantly reducing the number of model parameters and computational load while effectively enhancing the representation capacity of multi-scale pavement distress features. Second, a frequency-domain spatial attention is embedded in the S4 feature layer, where synergistic integration of frequency-domain filtering and spatial attention enables detail enhancement of distress edges and contours, automatically focuses on the distress regions, and suppresses background interference. The polarity-aware linear attention is incorporated into the S5 feature layer, by explicitly modeling polarity interactions, it effectively captures textural discrepancies between damaged regions and the intact road surface, and a learnable power function dynamically rescales attention weights to strengthen distress-specific feature responses. Finally, a cross-scale spatial feature fusion module (CSF^2^M) is developed to reconstruct and fuse multi-level spatial featurez, thereby improving detection robustness for pavement distresses with diverse morphologies under complex background conditions. Quantitative experiments indicate that, in contrast with the baseline RT-DETR, the presented framework improves the F1-score by 7.1% and mAP@50 by 9.0%, while reducing computational complexity and parameter quantity by 43.8% and 38.0%, respectively. These advantages enable LRD-DETR to be suitably deployed on resource-limited embedded platforms for real-time road distress detection.

## 1. Introduction

Environmental factors, sustained traffic loading, and other contributing elements inflict pavement damage of varying severity. Failure to quickly identify and remediate such problems can significantly shorten the service life of the highway, compromise the stability and safety of vehicle travel, and may even lead to serious traffic accidents. Consequently, the accurate identification and precise location of pavement distress types are of critical importance for effective road maintenance and traffic safety. Current pavement distress detection approaches include manual inspection, conventional image processing algorithms, and deep learning-based object detection techniques.

Manual inspection exhibits poor efficiency, requires substantial time and human resources, and remains easily affected by subjective bias. With the rapid expansion of highway networks, this conventional approach can no longer satisfy the inspection demands of large-scale road infrastructure. To achieve automated pavement distress detection, some researchers have introduced visual inspection technology, including threshold segmentation [1,2], edge detection [3], and other image processing algorithms [4]. Fernandez et al. [5] adopted a sequence of operations including logarithmic transformation, bilateral filtering, Canny detection, and morphological filtering to extract crack characteristics, and they then adopted a decision-tree heuristic strategy for crack classification. However, these methods have inherent limitations, lack robustness under complex environmental conditions, and a single algorithm struggles to detect multiple types of pavement distress, resulting in limited versatility and rendering them unsuitable for practical pavement distress detection scenarios.

Researchers have begun to combine image processing algorithms with machine learning to propose new detection methods. Hoang et al. [6] developed an automated asphalt pavement crack detection and classification model that combines these algorithms. Wu et al. [7] designed an automatic pothole detector by combining the pavement condition data collected by built-in vibration sensors on smartphones with machine learning classifiers. Zuo et al. [8] put forward CrackTree to tackle the challenges, including low contrast between cracks and backgrounds, non-uniform intensity distribution, and shadow disturbances. Sandra et al. [9] systematically assessed ten pre-trained CNN architectures for asphalt crack detection and classification tasks. Machine learning-based detection algorithms heavily rely on handcrafted features, and different types of pavement distress necessitate independently designed feature extraction processes, leading to poor flexibility and low efficiency.

Taking advantage of the advancement of deep learning technology, object detection based on deep learning has been applied in various fields. Currently, deep learning-based object detection algorithms primarily encompass one-stage models (e.g., single shot multibox detector (SSD) [10], you only look once (YOLO) [11,12,13]), two-stage models (e.g., R-CNN (Regions with CNN features) [14,15]), and transformer-based models (e.g., DETR (Detection Transformer) [16,17,18]). Applying object detection to pavement distress detection not only improves detection efficiency and accuracy but also enables real-time monitoring of pavement conditions, providing a scientific basis for road maintenance and management. To improve detection accuracy and speed, numerous researchers have proposed a series of enhancement strategies [19,20,21,22] for existing detection frameworks, including dataset augmentation, integration of attention mechanisms, adoption of lightweight network architectures, multi-scale feature fusion, and loss function optimization. Single-stage and two-stage detectors have long dominated computer vision tasks due to their mature technical architecture and excellent detection performance. Among them, YOLO-series models have become mainstream solutions due to their favorable speed–accuracy trade-off. However, such algorithms primarily rely on convolutional neural networks (CNNs) for feature extraction, which suffer from limited receptive fields that struggle to capture global features. Additionally, post-processing operations such as non-maximum suppression (NMS) introduce inevitable inference delays.

The Transformer-based DETR model [16] adopts an end-to-end design and has attracted industry attention to eliminate inference latency caused by components such as anchor boxes and NMS. Nevertheless, its high computational demands restrict the practical implementation. The advent of RT-DETR [23] marked a milestone as the first real-time end-to-end Transformer detector, surpassing contemporary YOLO variants in both speed and accuracy, and demonstrating significant application potential. Although RT-DETR effectively captures long-range feature dependencies among pavement distresses and provides a new technical pathway for intelligent inspection systems, it encounters numerous challenges in practical detection scenarios, including dense distress distribution, substantial scale variations (ranging from sub-pixel micro-cracks to large-area potholes), irregular shapes, and strong background interference (e.g., lane markings, shadows). To overcome these limitations, this work develops a lightweight RT-DETR-based detector for road distress detection. The core contributions are detailed as follows:

1. To address the high parameter count and limited feature representation capability of the RT-DETR backbone, we integrate the LFEModule into the C2f structure and incorporate the ADown adaptive down-sampling strategy, substantially decreasing the model’s parameter volume. During feature extraction, the LFEModule sequentially applies Gaussian smoothing and Scharr edge detection operators to enhance edge and texture representation while mitigating background interference and strengthening the model’s feature learning capabilities.
2. A frequency-space attention (FSA) is introduced in the S4 feature layer to capture local details and contextual information simultaneously. Through synergistic coordination of frequency-domain filtering and spatial attention, the system enhances the detail of the edge contours of road distress, adaptively focuses on target regions, and suppresses interference from complex pavement backgrounds (e.g., markings, shadows).
3. A polarity-aware linear attention mechanism (Pola) is adopted to redesign the AIFI module and apply it to the S5 feature layer. This mechanism captures texture discrepancies between distress regions and the background of the road surface through polarity interaction modeling and employs a learnable power function to dynamically rescale attention weights, significantly enhancing the representation of road surface distress features. This helps improve the detection precision of tiny distresses such as micro-cracks and small potholes.
4. A cross-scale spatial feature fusion module (CSF^2^M) is constructed based on the rectangular self-calibration module (RCM). By performing spatial reconstruction and fusion on multi-scale feature maps, this module enhances semantic expression in shallow-layer features and detailed characteristics of deep-layer features, strengthening the detection accuracy of the model for different kinds of road distress in complex backgrounds.

## 2. Related Work

This section elaborates on the key challenges and the corresponding improvement strategies within the domain of pavement distress detection. Precise discrimination of foreground features (e.g., cracks, potholes, and repair areas) from background features (e.g., normal pavement surfaces and lane markings) is essential for enhancing detection performance. This effectively reduces false-negative and false-positive rates, providing a significant reference value for highway maintenance. Several improvement methods have been developed to address these challenges. Zuo et al. [24] presented the Pavement-DETR model, which embeds the channel spatial shuffle (CSS) attention block into the S3 and S4 feature layers of the backbone network, allowing the model to focus more on pavement distress features. Additionally, they adopted a Conv3XC structure for feature fusion to further strengthen discrimination between foreground and background, and the experimental results showed that this model notably improves distress detection precision. Complex background noise, such as shadows, water stains, and changes in illumination on the road surface, can severely interfere with the precision and computational efficiency of detection. Therefore, Liu et al. [25] proposed the Crack-DETR model. It incorporates a multi-frequency feature extraction and fusion module (MFEF), which uses attention mechanisms from high-frequency and low-frequency perspectives to enhance pavement crack features and suppress background noise, achieving excellent performance in crack detection. Radzi et al. [26] proposed RT-DETR-Pothole to achieve high-performance pothole detection for small or obscured potholes, which are easily overlooked in actual scenarios. They achieved scores mAP@0.5 and mAP@0.5:0.95 of 83.4% and 56.5%, respectively, while maintaining a shorter inference time. The irregularity in the scale and orientation of the distresses (e.g., cracks and potholes with diverse forms), the masking effect of complex backgrounds (e.g., pavement textures and markings), and the high similarity between some distresses and background further constrain the improvement of detection accuracy. Pan et al. [27] proposed a real-time dynamic scale-aware fusion detector termed RT-DSAFDet, which integrates a flexible attention (FA) block adaptive to defect shapes and backgrounds, a DASF module for multi-scale feature fusion, and an efficient sampling down (SD) module. This structure strengthens multi-scale feature extraction and fusion while greatly reducing model parameters and computation. Han et al. [28] proposed an efficient focus real-time pavement distress detector named EF-RT-DETR. Its backbone network captures fine-grained features, a feature focusing module reduces noise impact and enhances fine-grained feature extraction, and a lightweight multi-scale fusion module facilitates integration of regional and holistic information. This model significantly reduces background false detection rates while improving detection speed and accuracy.

The parameter count and computational complexity of the network architecture exert considerable influence on the pavement distress detection task. Overabundant parameters compromise deployment practicality, while elevated computational demands result in delayed response times, hindering compliance with real-time processing constraints Chen et al. [29] proposed a lightweight real-time detection model (L-RTDETR) to balance accuracy and speed. This framework employs a large selective kernel network (LSKNet) as the backbone to construct a lightweight distress detection network, reducing the size and parameters of the network. Implements intra-scale feature interaction based on deformable attention (DAttention), extracting contextual information via a sparse attention mechanism. Furthermore, a novel loss function was introduced to address sample imbalance and enhance small-target detection capability. Experiments showed that L-RTDETR achieves a 35.8% reduction in parameters and reaches 61.6 FPS. Wang et al. [30] developed a UAV image-based pavement distress detection model that was improved from RT-DETR. It utilizes the RepNCSPELAN module to capture multi-scale contextual information, and it augments feature heterogeneity and representational capacity via efficient hierarchical feature integration. The Adown module was used to reduce spatial dimensionality during down-sampling. Experiments indicate that the model reduces parameters and computation by 54.4% and 53.5%, respectively. Furthermore, some researchers have explored new models that combine the advantages of CNN and Transformer. For example, the fast pavement distress detection network (FPDDN) proposed by Zhang et al. [31] integrates Transformer, D2f and SFB modules. Among them, the SFB module can amplify the information weights of small targets during feature fusion. D2f, based on depth-wise separable convolution, aims to compress the network and accelerate inference. The deformable attention mechanism empowers the FPDDN with robustness against morphological distortions in pavement distresses, it improves the recognition precision for irregular distresses.

Although the above models have made progress in speed, accuracy, and lightweight design, they still face many challenges in practical application scenarios. Improving the detection sensitivity for subtle cracks, effectively suppressing interference from complex pavement backgrounds (e.g., textures and markings), and further optimizing model efficiency to meet real-time inspection demands in large-scale road maintenance scenarios are still significant directions for future research. This work presents the LRD-DETR model, which improves upon the RT-DETR framework in the following three key ways: first, by constructing a lightweight backbone network; second, by introducing an attention mechanism; and third, by designing a cross-scale feature integration module. These improvements enable the model to better satisfy the inspection demands of multi-class pavement distress.

## 3. Materials and Methods

### 3.1. LRD-DETR Overall Structure

The backbone network is responsible for extracting hierarchical feature maps at different scales from the source imagery. The feature fusion network then integrates and enhances these features derived from the backbone, optimizing feature representation to improve the model’s detection capability across various scales. For detection tasks characterized by complex road surface backgrounds, various types of distress, and significant scale variations, the limited feature learning ability of the original backbone adversely affects the final detection performance. Meanwhile, the volume of model parameters and computational overhead are also critical factors for practical deployment.

**Figure 1:**
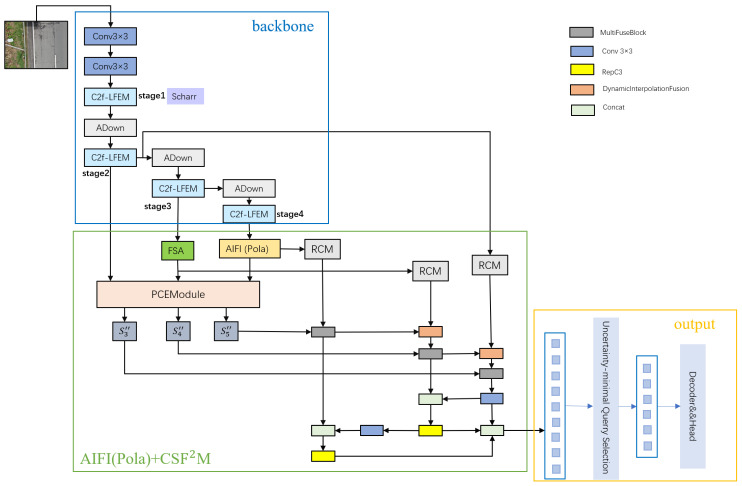
Architecture of LRD-DETR.

To overcome these problems, this study proposes a novel road distress detection framework. First, the LFEModule [32] was integrated into the C2f architecture and combined with ADown [33] to form a lightweight backbone network. Second, applying the frequency spatial attention (FSA) module [34] and polar-aware linear attention [35] (Pola) to mid- and high-level feature maps, respectively, can collaboratively improve feature representation capabilities at different semantic levels. Specifically, FSA operates on mid-level features, focusing on local detail enhancement and noise suppression, effectively highlighting key structural characteristics of pavement distress. Meanwhile, the Pola mechanism is applied to high-level features, leveraging its polarity-aware modeling capability to strengthen global semantic associations and contextual interactions. The synergistic integration of these two approaches not only enhances the precision of the model in identifying and localizing pavement distress regions, but also substantially strengthens its stability and adaptability in complex scenarios. Finally, by introducing a rectangular self-calibration module (RCM) [36] to reconstruct multi-scale spatial feature maps that are rich in disease detail information. On this basis, through a cross-scale feature fusion strategy, semantic and detail information at different levels are effectively integrated, thereby alleviating the problem of limited detection performance of multi-scale targets (such as distresses of different sizes) due to the limited representation ability of single-scale features, providing higher-quality spatial feature maps for the detection head. The complete architecture and its details of implementation are illustrated in Figure 1.

### 3.2. Lightweight Backbone Network

ResNet18, serving as the primary network component of the RT-DETR model, is constrained by its shallow network architecture, leading to restricted feature representation capabilities, especially in the context of multi-scale object detection tasks. This limitation can potentially compromise the model’s detection efficacy. The expansion of network depth has been demonstrated to augment the model’s feature representation capability. However, this expansion concomitantly leads to a significant increase in the model’s parametric volume and computational overhead. In the context of road distress detection, our objective is to achieve an optimal trade-off between detection precision and inference efficiency. This involves realizing inference in real-time while preserving superior detection fidelity. To address these problems, this work presents a newly developed backbone framework.

**Figure 2:**
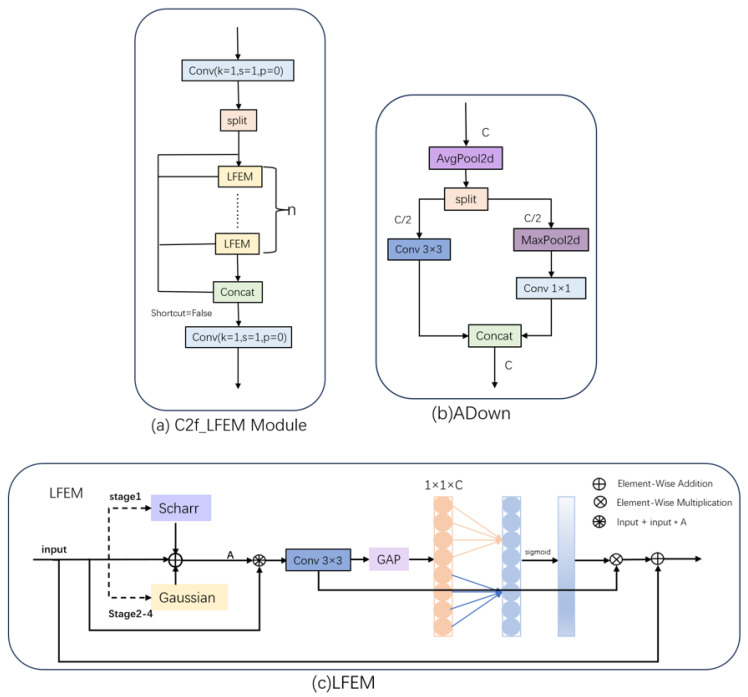
Schematic diagram of the backbone architecture, (**a**,**b**) represent C2f-LFEM and ADown of the backbone network, respectively; (**c**) represents LFEModule of C2f-LFEM.

ADown is a down-sampling convolution block (its structure is shown in Figure 2b) from YOLOv9 [33], and its operation process is as follows. First, the average pooling operation is conducted on the input features to retain the global detail information; subsequently, a dual-branch parallel processing architecture is entered. One branch captures local spatial patterns via convolutional layers, whereas the other adopts max-pooling operations to highlight the key features in the feature map. Finally, the feature maps from dual branches are integrated at different scales to achieve multi-scale feature aggregation. The module is applied to the pavement distress detection model, which helps to enhance the discriminative capacity of the model for distress types with significant morphological differences. Furthermore, dual-branch processing can reduce the parameter scale while maintaining the rich features, which provides the possibility for the deployment of the model on edge devices.

The LFE Module (as shown in Figure 2c) adaptively captures salient features from the input feature map $F_{in}$ via the following selection mechanism (Equation (1)):

(1) $$A=\left\{\begin{array}{cc} A_{edge}\left(F_{in}\right), & \mathrm{stage}=1\\ A_{gauss}\left(F_{in}\right), & \mathrm{stage}=2,3,4 \end{array}\right.$$

where $A_{edge}$ represents the edge features, and $A_{gauss}$ represents the Gaussian features. The shallow feature map (stage 1) retains abundant edge and high-frequency information that is crucial to the target location. Using the Scharr operator (as shown in Equation (2)) to improve edge features, it helps the model distinguish between distressed regions and the normal road surface, improving the localization accuracy of small targets (e.g., tiny cracks).

(2) $$S_{x}=\left[\begin{array}{ccc} -3 & 0 & 3\\ -10 & 0 & 10\\ -3 & 0 & 3 \end{array}\right]\;S_{y}=\left[\begin{array}{ccc} -3 & -10 & -3\\ 0 & 0 & 0\\ 3 & 10 & 3 \end{array}\right]$$

Scharr filters were integrated into the convolutional layers ${Conv}_{2D}^{S_{x}}$ and ${Conv}_{2D}^{S_{y}}$, allowing them to process $F_{in}$ along the horizontal and vertical axes, respectively. The edge responses from the two axes are combined to obtain the final edge intensity value $A_{edge}$, as shown in Equation (3).

(3) $$A_{edge}=\sqrt{{\left({Conv}_{2D}^{S_{x}}\left(F_{in}\right)\right)}^{2}+{\left({Conv}_{2D}^{S_{y}}\left(F_{in}\right)\right)}^{2}}$$

In deep feature maps (stages 2, 3, and 4), edge information weakens as the feature dimension decreases. Using a fixed Gaussian kernel (as expressed in Equation (4)) can enhance the salient features of pavement distress (e.g., contours of potholes and repairs), while suppressing background noise. This hierarchical processing significantly enhances the feature learning capability of the model.

(4) $$A_{gauss}=G_{\sigma }^{k\times k}\left(x\right)=\frac{1}{2\pi \sigma^{2}}e^{-\frac{x^{2}+y^{2}}{2\sigma^{2}}}$$

Integration and enhancement of the feature were achieved through Equation (5). This method effectively preserves the spatial characteristics of the original features $F_{in}$, while simultaneously incorporating the structural information of the Edges or Gaussian features, consequently strengthening the model’s capacity for feature representation.

(5) $$F_{out}={Conv}_{2D}^{3\times 3}\left(\left(F_{in}⊗A\right)⊕F_{in}\right)$$

Finally, channel weights are adjusted through efficient channel attention (ECA) to highlight the disease characteristics.

(6) $$F_{out}=Norm\left(sigmoid\left({Conv}_{2D}^{k}\left(GAP\left(F_{out}\right)\right)\right)⊗A\right)⊕F_{in}$$

The depth-wise separable convolutions and 1 × 1 convolutions utilized in the LFEM form the lightweight convolutional structures. We integrate LFEM into the C2f framework as shown in Figure 2a, which can simultaneously facilitate efficient extraction of pavement distress features and significantly decrease the model’s parameter volume. This design achieves model lightweighting while preserving the performance of feature extraction. The empirical findings presented in Table 1 further validate the effectiveness of the proposed backbone network.

### 3.3. Strengthen Road Distress Features and Weaken Background Interference

In real-world road distress detection scenarios, cameras are highly susceptible to factors such as changes in lighting, road shadows, water stains, oil stains, traffic lane markings, and vehicle obstructions, resulting in incomplete capture of pavement distress feature information. Furthermore, road distresses exhibit uneven spatial distribution (e.g., longitudinal and transverse cracks may span multiple areas of an image). These challenges may result in omission errors or misclassifications during subsequent detection procedures. To mitigate these issues, this study incorporates an attention mechanism to strengthen the model’s feature extraction capacity, enabling it to accurately identify the type and location of pavement distresses within complex backgrounds.

**Figure 3:**
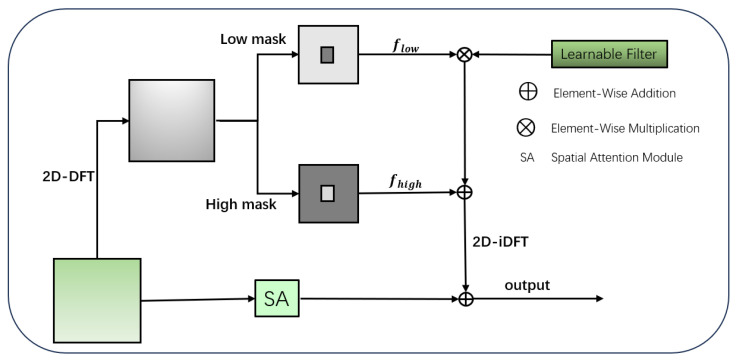
Structural diagram of the FSA module.

For missed detections or false positives of pavement distress features caused by illumination variations and complex background interference (e.g., road markings and shadow artifacts), this paper incorporates frequency-space attention (FSA) [34] in the intermediate feature layer S4, as depicted in Figure 3. This mechanism converts the feature representation into the frequency domain for processing to capture global contextual information that is challenging to acquire through spatial-domain analysis alone, and it subsequently fuses it with fine-grained spatial features. This dual-domain complementary strategy enhances the model’s feature representation capacity.

The spatial-domain feature map ${\hat{F}}_{i}^{\prime }\in R^{H\times W\times C}$ is converted into the frequency domain feature map $f_{i}\in R^{H\times W\times C}$, through a 2D-DFT (two-dimensional Fourier transform), as described in Equation (7).

(7) $$f_{i}\left(U,V\right)=\sum_{x=0}^{H-1}\sum_{y=0}^{W-1}{\hat{F}}_{i}^{\prime }\left(x,y\right)e^{-j2\pi \left(\frac{U_{x}}{H}+\frac{V_{y}}{W}\right)}$$

where ${\hat{F}}_{i}^{\prime }\left(x,y\right)$ indicates the spatial-domain pixel intensity of the original feature representation, and H and W correspond to the vertical and horizontal dimensions of the feature map, respectively.

In the frequency domain, an image is decomposed into a superposition of different frequency components. The low-frequency components correspond to regions with relatively smooth gray-scale changes (a large region of road background), while the high-frequency components correspond to regions with sharp gray-scale variations (contours of pavement distresses such as cracks, potholes, and repairs). The frequency response is dynamically modulated by constructing a high-frequency mask $M_{high}\in R^{H\times W\times 1}$ (which enhances the high-frequency components while suppressing the low-frequency components) and a low-frequency mask $M_{low}\in R^{H\times W\times 1}$ (which enhances the low-frequency components while suppressing the high-frequency components), as shown in Equations (8) and (9).

(8) $$f_{i,low}=f_{i}⊗M_{low}$$

(9) $$f_{i,high}=f_{i}⊗M_{high}$$

where ⨂ denotes the element multiplication, and $f_{i,high}$ and $f_{i,low}$ represent the high-frequency and low-frequency features, respectively.

In extensive pavement regions, both interference factors (e.g., illumination variations, water stains, and oil stains) and distress features (e.g., crack contours and pothole edges) manifest themselves as high-frequency components. The former can be suppressed by using a low-frequency mask to reduce high-frequency noise interference, while the latter can be enhanced by using a high-frequency mask. This frequency-aware attention mechanism effectively amplifies the frequency disparity between the pavement distress and interference regions.

For $f_{i,low}$, an adaptive global filter $N\in R^{H\times W\times C}$ was applied to perform filtering. which helps to reduce the impact of road surface noise on detection accuracy. Then, these features are fused with high-frequency features, as shown in Equation (10), where ⊕ denotes element-wise addition.

(10) $$f_{i}^{\prime }=f_{i,high}⊕\left(f_{i,low}⊗N\right)$$

Finally, the features are restored to a spatial-domain feature using a 2D-iDFT (two-dimensional inverse Fourier transform), as formulated in Equation (11).

(11) $${\hat{F}}_{i}^{\prime \prime }\left(x,y\right)=\frac{1}{HW}\sum_{U=0}^{H-1}\sum_{V=0}^{W-1}f_{i}^{\prime }\left(U,V\right)e^{j2\pi \left(\frac{U_{x}}{H}+\frac{V_{y}}{W}\right)}$$

(12) $${\hat{F}}_{i}out={\hat{F}}_{i}^{\prime \prime }⊕SA\left({\hat{F}}_{i}^{\prime }\right)$$

In the spatial domain, the SA module employs channel-wise average pooling and maximum pooling to generate spatial attention maps, enabling focusing on distress regions (e.g., the linear structure of cracks and the edge contours of potholes) while weakening the feature responses of interfering regions like water stain and oil patches. The feature fusion of the spatial and frequency domains is illustrated in Equation (12).

As a pivotal hub bridging shallow-level details and deep-level semantics, the intermediate feature layer, subsequent to frequency domain processing via frequency domain spatial attention, can effectively enhance global structural information, suppress redundant noise interference, and amplify the feature response at disease edges. Through synergistic fusion through integration with the spatial attention mechanism, feature representation achieves both global semantic consistency and local detail precision, mitigating the fragmentation of crack features induced by limited local receptive fields. Figure 3 illustrates the architecture of the FSA module. As demonstrated by the comparative data summarized in Table 2, incorporation of FSA into the S4 feature layer can improve the model’s detection performance with minimal additional parameter overhead.

**Figure 4:**
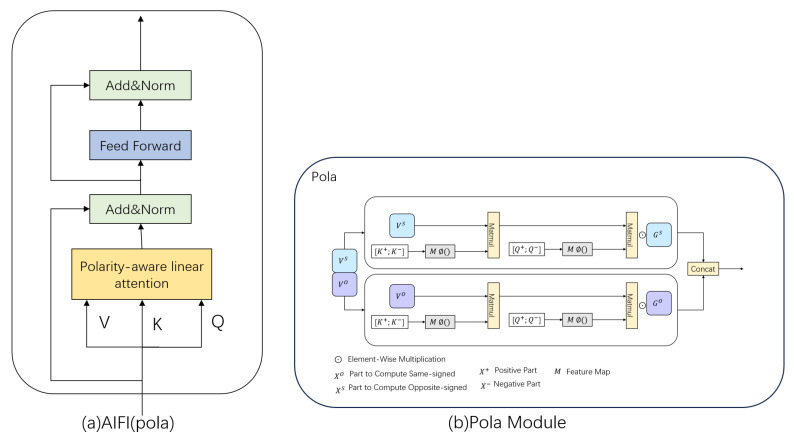
Structural diagram of the AIFI block; (**a**) represents replacing the multi-head attention mechanism in AIFI with the polarity-aware attention mechanism, and (**b**) represents the structure of the polarity-aware attention mechanism.

In the RT-DETR model, the AIFI module serves as an efficient intra-scale feature interactor, employing the self-attention mechanism only in high-level feature maps rich in semantic information for global context modeling. However, when dealing with a large number of high-resolution disease images, its quadratic computational complexity significantly increases the computational burden, and traditional linear approximations may lead to the loss of context information. To solve this problem, a polarity-aware linear attention mechanism (Pola) [35] is introduced to reconstruct the AIFI module (as shown in Figure 4a), which lowers the computational complexity of self-attention from quadratic to linear order, markedly enhancing computational performance. Meanwhile, this mechanism explicitly models the complete interactions between positive and negative polarities, generating attention maps that are more discriminative and closer in distribution to those produced by Softmax attention, which helps to enhance the localization accuracy of pavement distress regions. The Pola module is illustrated in Figure 4b.

The Pola mechanism adopts a polar separation approach to divide the query and key into positive and negative polarity components, as shown in Equation (13). The inner product between the query and the key can be divided into four parts, as shown in Equation (14).

(13) $$q=q^{+}-q^{-}\;k=k^{+}-k^{-}$$

(14) $$<q,k>=<q^{+},k^{+}>+<q^{-},k^{-}>-<q^{+},k^{-}>-<q^{-},k^{+}>$$

By separately calculating the interactions of same-sign (first two terms) and opposite-sign (last two terms) components, to completely retain the information of all polarity combinations and avoid the problem of reduced expression power caused by the inability to fully capture the complex relationships among various types of road distress. This approach of polarity interaction modeling allows for a more complete capture of the textural differences between the distress regions and the background of the road surface, offering distinct advantages for the detection of long cracks.

The kernel function is applied to map features into a higher-dimensional space to reduce computational complexity, and attention weights are calculated using Equation (15).

(15) $$M\left(q,k^{T}\right)\approx \;\left(\phi \left(q^{+}\right){\phi \left(k^{+}\right)}^{T}+\phi \left(q^{-}\right){\phi \left(k^{-}\right)}^{T}\right)-\left(\phi \left(q^{+}\right){\phi \left(k^{-}\right)}^{T}+\phi \left(q^{-}\right){\phi \left(k^{+}\right)}^{T}\right)$$

Each value vector $v\in R^{N\times d}$ is split into two complementary halves across the channel axis, denoted as $v^{s}$ and $v^{o}$. These are used to process the responses of the same-sign and opposite-sign, respectively. The final output is obtained by weighting and combining these two components using two learned polarity-aware coefficient matrices, $G^{s}\in R^{N\times \frac{d}{2}}$ and $G^{o}\in R^{N\times \frac{d}{2}}$, as expressed in Equation (16). This allows the model to adaptively learn the complementary relationship between same-sign and opposite-sign information.

(16) $$O_{t}=\left[\frac{Ø\left(\left[q_{t}^{+};q_{t}^{-}\right]\right)\sum_{i=1}^{N}{Ø\left(\left[k_{i}^{+};k_{i}^{-}\right]\right)}^{T}v_{i}^{s}}{Ø\left(\left[q_{t}^{+};q_{t}^{-}\right]\right)\sum_{j=1}^{N}{Ø\left(\left[k_{j}^{+};k_{j}^{-}\right]\right)}^{T}}⊙G^{s};\frac{Ø\left(\left[q_{t}^{+};q_{t}^{-}\right]\right)\sum_{i=1}^{N}{Ø\left(\left[k_{i}^{-};k_{i}^{+}\right]\right)}^{T}v_{i}^{o}}{Ø\left(\left[q_{t}^{+};q_{t}^{-}\right]\right)\sum_{j=1}^{N}{Ø\left(\left[k_{j}^{-};k_{j}^{+}\right]\right)}^{T}}⊙G^{o}\right]$$

where ⊙ denotes the element-wise multiplication.

To restore low-entropy features similar to those of Softmax, a learnable channel-level power function is employed to amplify strong responses and suppress weak ones, thereby reducing the entropy of the attention distribution. This facilitates a more accurate concentration of attention weights on damage regions, effectively suppressing interference from the road surface background, and thus improving the accuracy of pavement distress localization.

### 3.4. Cross-Scale Spatial Feature Fusion Module

Pavement distresses manifest as substantial scale variations (e.g., micro-cracks are only a few pixels wide, while transverse crack, longitudinal crack, and potholes may occupy hundreds of pixels). Single-scale feature extraction may miss extremely small or large distress instances, leaving road maintenance departments unable to grasp the real condition of road surface distress. If not repaired in time, it will accelerate the deterioration, affect the service life of the roadway, and even increase the risk of severe traffic incidents. Furthermore, feature maps at varying scales exhibit distinct receptive fields. Small-scale feature maps emphasize the analysis of minute features (e.g., tiny cracks and potholes), while large-scale feature maps facilitate the comprehension of the correlation between distress regions and the road surface background (e.g., the relative position of cracks and pavement). Fusing multi-scale features can effectively address the problems posed by significant scale variations among distresses and complex background interference.

**Figure 5:**
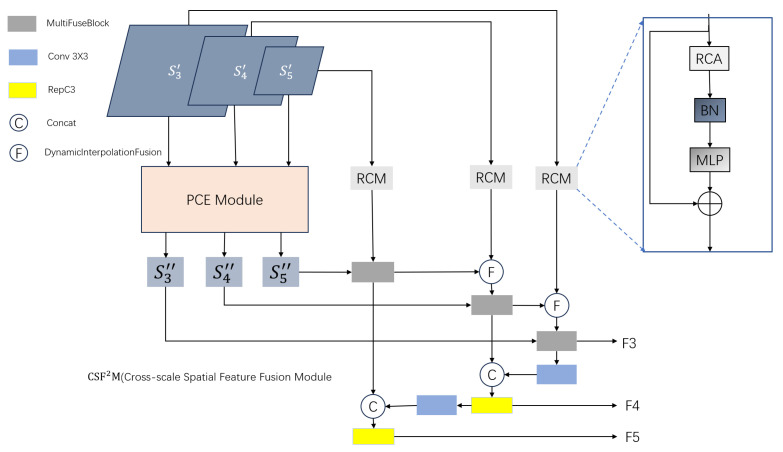
Structure diagram of CSF^2^M module.

This paper proposes a cross-scale spatial feature fusion module (CSF^2^M), as illustrated in Figure 5, uses a “top-down guidance + bidirectional feature complement” fusion strategy. The module first extracts and fuses multi-scale feature maps through the pyramid context extraction (PCE) module. Specifically, the different scale feature maps $S3^{\prime }$, $S4^{\prime }$, and $S5^{\prime }$ with resolutions of 1/8, 1/16, and 1/32 respectively were adjusted to feature maps of fixed size by the average pooling, which were then concatenated along the channel dimension to generate pyramid F5. Subsequently, three stacked RCM modules [36] were used for pyramid feature interaction and to extract multi-scale feature information. Finally, the features are split and up-sampled on the original scale, generating new feature maps $S3^{\prime \prime }$, $S4^{\prime \prime }$, and $S5^{\prime \prime }$ and spatial feature reconstruction. To make the encoder features pay more attention to pavement distress, RCM is used to reconstruct spatial features. In particular, high-level spatial features of the PCE module are fused with low-level spatial features of the corresponding scale using dynamic interpolation fusion and multi-feature fusion blocks to form new spatial features. Finally, RepC3 further integrates two adjacent spatial features. RepC3 contains two 1 × 1 convolutions to adjust the number of channels, and N RepBlocks composed of RepConv are used for feature fusion.

The RCM consists of a rectangular self-calibration attention, Batch Normal, and MLP. For the input feature map, RCA applies adaptive average pooling to the horizontal and vertical directions of the input feature map, which is represented as $x\in R^{H\times W\times C}$. We obtain the two axial vectors $H_{P}\left(x\right)$ and $V_{P}\left(x\right)$, and their summation forms a rectangular region that focuses on characteristics of the distress. The shape self-calibration function is then designed to calibrate this rectangular region, as expressed in Equation (17).

(17) $$\xi_{C}\left(\bar{y}\right)=\delta \left(\psi_{k\times 1}\left(\phi \left(\psi_{1\times k}\left(\bar{y}\right)\right)\right)\right)$$

where $\psi_{1\times k}$ represents the large-kernel strip convolution, k denotes the size of the convolution kernel, ϕ denotes normal and non-linearity (BN + ReLU), and δ denotes the sigmoid function.

Pavement distresses have directionality (e.g., transverse, longitudinal, and oblique cracks). The RCA module can adaptively capture disease characteristics in different directions, improving the sensitivity of the model to crack orientations. Furthermore, a feature fusion function is designed to fuse attention features with input features, as depicted in Equation (18).

(18) $$\xi_{F}\left(x,y\right)=\psi_{3\times 3}\left(x\right)⊙y$$

where y represents the calibrated attention feature, $\psi_{3\times 3}$ denotes a depth-wise convolution used to extract local details of the input feature, and ⊙ denotes element-wise multiplication (Hadamard product) for feature fusion.

The RCM output can be expressed using Equation (19).

(19) $$F_{out}=\rho \left(\xi_{F}\left(x,\xi_{C}\left(H_{P}\left(x\right)⊕V_{P}\left(x\right)\right)\right)\right)+x$$

where ρ refers to BN and MLP, and ⊕ denotes broadcast addition.

Constructing a cross-scale spatial feature fusion module during the feature fusion stage can effectively address issues such as interference from complex road background interference, significant scale variations across distress instances, and missed detections of subtle distresses. Specifically, for pavement distress features with significant scale differences, the pyramid context extraction module can simultaneously capture global features (e.g., large-area distress regions such as patches, cracks, and potholes) and local features (e.g., initiation or termination points of individual cracks) from the image, providing more comprehensive scale information for subsequent spatial feature reconstruction and fusion. Second, fine cracks in shallow feature maps are prone to missed or false detections due to ambiguous morphological patterns. The shape self-calibration function of the rectangular self-calibration module adaptively focuses on the crack region, suppressing road surface background interference, thereby improving the detection accuracy of minute distresses. Finally, during cross-scale feature fusion (combining deep semantic features with shallow detail features), the model leverages semantic information provided by the pyramid context extraction module to dynamically determine regions requiring detail enhancement versus background suppression. This ensures that the fused features represent the boundaries and internal structures of pavement distress more clearly and accurately.

## 4. Results and Analysis

### 4.1. Experimental Environment and Parameter Configuration

All experiments were conducted with identical hardware and software configurations to ensure a fair comparative evaluation of the results. The operating system was Windows 11, with an Intel (R) Core (TM) i9-14900HX CPU, NVIDIA GeForce RTX 4060 GPU and 32 GB of RAM. The Python version used was 3.10, and the Torch and Torchvision versions were 2.1.1+cu118 and 0.16.1+cu118, respectively.

The training parameters were set as follows: the batch size and workers were set to 8 and 4 (limited by the GPU memory (16 GB), as a batch size of 8 can ensure the stability of model training while improving training efficiency; 4 workers are set to match the batch size, accelerating data loading and improving training speed), respectively; the AdamW optimizer was adopted (compared with other optimizers (e.g., SGD, Adam), AdamW has better adaptability to Transformer-based models, which can effectively improve the stability of model training); the size of all input images was set to 640 × 640 pixels (this size balances the feature extraction accuracy and model inference speed, which can fully retain the details of road stress features (e.g., small cracks) while ensuring that the model can run in real time on edge devices); the initial learning rate of 0.0001 (a lower initial learning rate is selected to avoid gradient oscillation and ensure stable convergence of the model); and weight decay of 0.0001 and momentum of 0.9 (weight decay is used to suppress model overfitting by regularizing the model parameters, and a momentum of 0.9 is the default value). The model was trained for 500 epochs (the model convergence curve shows that the loss value tends to stabilize after 500 epochs, and continuing training will lead to overfitting).

### 4.2. Dataset and Evaluation Indicators

Comprising 2,440 high-resolution images (2592 × 1944 pixels) acquired by unmanned aerial vehicles (UAV) in diverse road infrastructure in China, the UAV-PDD2023 dataset [37] covers more than 11,150 annotated pavement distress instances. The collection spans three road classifications—highways, provincial roads, and county roads—and captures surface conditions under the following two distinct weather regimes: clear weather and post-rainfall scenarios (within one hour of precipitation). Six canonical pavement distress types were systematically annotated, namely, longitudinal cracks, transverse cracks, oblique cracks, alligator cracks, repairs, and potholes, which were subsequently divided into training, validation, and test subsets in a proportion of 7:1:2.

**Figure 6:**
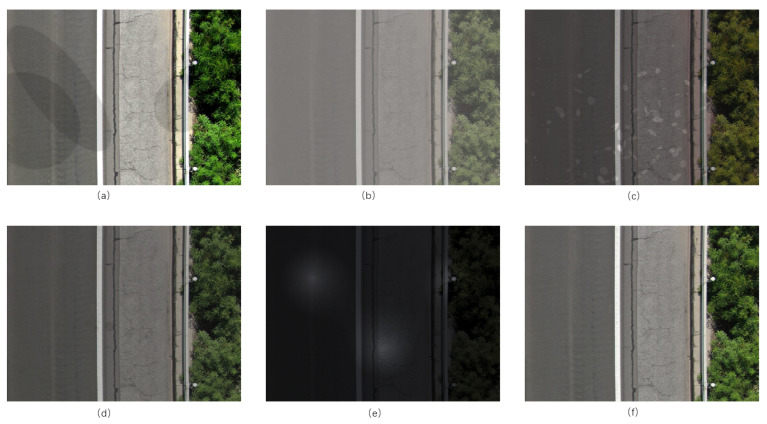
M-PDD: (**a**) illustrates the condition under direct strong light; (**b**) shows a foggy day; (**c**) presents a rainy day; (**d**) displays the condition with stains; (**e**) depicts night lighting conditions; and (**f**) represents the pavement condition under normal lighting.

To further enhance the robustness of the pavement distress detection model in complex real-world scenarios, a new diverse pavement distress dataset M-PDD, based on the public dataset UAV-PDD2023, was used. Selected instances from the M-PDD dataset are presented in Figure 6. Figure 6a illustrates the condition under strong direct light; Figure 6b shows a foggy day; Figure 6c presents a rainy day; Figure 6d displays the condition with stains; Figure 6e depicts the conditions of light at night; and Figure 6f represents the condition of the pavement under normal light. This dataset combines newly generated synthetic images with real pavement damage images collected in the original UAV-PDD2023 dataset in a 1:1 ratio to form the final pavement distress dataset. This approach of combining real data with synthetic data not only retains the characteristics of pavement damage in the real world, but it also introduces a variety of environmental factors, providing more comprehensive learning materials for model training.

The newly generated synthetic images were produced through StyleGAN2 (to generate small-sample disease slices, including potholes, alligator cracks, and repair) and image fusion algorithms. To perform a comprehensive assessment of the generated image quality, we adopt the following four metrics for phased validation: during the distress slice generation phase, the structural similarity index measure (SSIM) and the Fréchet Inception Distance (FID) are used to quantify the visual fidelity and distribution authenticity of the generated slices (results shown in Table 1). Higher SSIM values (approaching 1) and lower FID scores indicate superior structural similarity and alignment of the feature distribution between generated and real images, indicating a higher quality of generated images.

During the image fusion stage, we use Balance and Spatial metrics to evaluate the distribution equilibrium of distress categories and the rationality of spatial layout in the fused images (results shown in Table 2). The fusion process involves mixing slices of transverse cracks, longitudinal cracks, and network cracks with data-enhanced slices of potholes, repairs, and alligator cracks before embedding them in the pavement background. The balance score closer to 1, indicates a more balanced distribution of samples across different pavement distress categories in the synthetic images, while the spatial score approaching 1 signifies a more reasonable spatial layout of distress regions.

In order to conduct a thorough evaluation of the detection capabilities of the proposed model, this study performed comparative experiments with other state-of-the-art detection architectures using the same dataset. The evaluation metrics for the experimental results were categorized as accuracy and speed. The accuracy metrics mainly encompass Precision, Recall, F1-score (the harmonic mean of precision and recall), and mean average precision (mAP). For speed metrics, frames per second (FPS) and Giga floating point operations per second (GFLOPs) were adopted.

Precision: Measures the model’s ability to correctly identify target objects. Higher precision indicates a lower false-positive rate.

(20) $$P=\frac{TP}{TP+FP}$$

Recall: Quantifies the model’s ability to recognize true positive instances. A higher recall signifies a lower false-negative rate.

(21) $$R=\frac{TP}{TP+FN}$$

F1-score: Comprehensively considers precision and recall, balancing the model’s performance in terms of missed and false detections.

(22) $$F1=2\frac{P\times R}{P+R}$$

mAP: The average value of AP (Average Precision) in all categories, where AP represents the area under the precision–recall curve.

(23) $$mAP=\frac{\sum_{i=1}^{N}\int_{0}^{1}P_{i}\left(R\right)dR}{N}$$

FPS (Frames Per Second): Represents the throughput of images processed per second, reflecting real-time inference capabilities. A higher FPS denotes a faster detection speed.

### 4.3. Ablation Experiments

To confirm the efficacy of the proposed approach in the detection of pavement distress, this study conducted extensive ablation experiments. RT-DETR incorporates a backbone network, an efficient hybrid encoder, and a decoder augmented with auxiliary prediction heads. All experimental evaluations were conducted using the UAV-PDD2023 dataset in consistent configurations, with the corresponding results presented in Table 3. We apply the following single-variable principle: while keeping all other modules constant, individually evaluating the influence of each component on the object detection model’s performance metrics Without any improvement modules, the baseline (RT-DETR) achieved precision (P), recall (R), and mAP@0.5 of 88.4%, 74.5%, and 79.6%, respectively.

When only the improved backbone network (C2f-LFEM+ADown) was used, compared with the baseline model, the parameters and GFLOPs of the model were reduced by 28.9% and 39.8%, respectively. This result demonstrates the efficacy of the proposed backbone network design in achieving a lightweight structure and increasing computational efficiency. When only the FSA module is used in layer S4, P, R, and mAP@0.5 showed improvements of 3.7%, 2.3%, and 3.5%, respectively. And when only the attention mechanism in the AIFI module was replaced with Pola. P, R, and mAP@0.5 showed improvements of 1.8%, 3.2%, and 4.6%, respectively. As evidenced by the results in Table 3, each improved module has enhanced the efficacy of pavement distress detection to varying degrees in regard to both detection accuracy and lightweight design, fully proving the validation of the improvements made to each module.

In order to further confirm the compatibility and collaborative effects among the different improved modules, this research carried out combination experiments by merging multiple improved modules, with the objective of thoroughly evaluating their collective performance post-integration. As can be seen from the findings in Table 3, after introducing the Pola and FSA mechanisms, the detection model maintains stable parameter count and computational complexity while achieving remarkable progress in detection precision and recall, demonstrating the competence of the proposed modules in suppressing background interference and enhancing pavement distress feature representation. Combining the improved backbone network with the polar-aware linear attention (Pola), P, R, and mAP@0.5 showed improvements of 0.7%, 5.5%, and 5.2%, respectively, while parameter size was reduced by 28.4% and computational complexity by 38.9%. The results show that the new backbone network and the polar-aware attention mechanism are compatible. Building on the combination of the improved backbone network and the Pola, the CCFM was replaced with the CSF^2^M. P, R, and mAP@0.5 showed improvements of 4.2%, 3.9%, and 4.9%,respectively, compared to the benchmark model. The findings demonstrate that reconstructing and fusing multi-scale spatial features effectively enhances the representation of pavement distress details while reducing the missed detection rate.

Moreover, based on the use of the improved module, spatial frequency attention (FSA) was applied to the feature layers S3 and S4, as presented in Table 4. When FSA was used in the S3 feature layer, P, Rm and mAP@0.5 showed corresponding values of 88.9%, 78.6%, and 84.2%, respectively. The P, R, and mAP@0.5 when using FSA in the S4 feature layer were 91.5%, 83.6%, and 85.8%, respectively. Based on experimental results, incorporating the FSA module into the S4 feature layer yields a significantly better detection performance than S3; consequently, this study employed the FSA module exclusively in the S4 feature layer.

Finally, this study systematically integrates and merges all modules to construct a complete model architecture. Through comprehensive comparative experiments and performance tests, the outcomes reveal that P, R, and mAP@0.5 achieved gains of 8.9%, 8.3%, and 9.0%, respectively, compared to the benchmark model, while parameters and GFLOPs were reduced by 43.8% and 38.0%, respectively. This study confirms that this optimized model architecture enables effective enhancement of both precision and recall in road distress detection, while significantly reducing the number of model parameters and computational load, making it suitable for deployment on edge devices. Consequently, this integrated approach achieves effective collaboration among modules and yields remarkable progress across multiple evaluation metrics, fully validating the overall effectiveness and practical value of the proposed improvement strategy.

This conclusion is further supported by the visualization results of the heatmaps, illustrating that, compared to the baseline model, LRD-DETR focuses more attention on the pavement distress regions, improving the salience of distress features while successfully minimizing background interference, thereby proving the effectiveness of the proposed modules in strengthening the discriminative capacity of the model.

### 4.4. Comparison Experiments

This study compared the improved model with several main object detection algorithms, including the YOLO series and DETR variants. Specifically, experiments were conducted using the UAV-PDD2023 dataset, as detailed in Table 5. To assess the efficacy of the models, identical parameter configurations were applied in the training and testing phases of each model. The evaluation results confirm that our model provides superior performance compared to the current state-of-the-art object detection methods in precision (P), recall (R), and mAP@0.5, indicating that the proposed model has a good future application for the detection of pavement distresses. The detection results of Pavement-DETR in Table 5 were obtained from [24]. To confirm the functionality of the developed models, comparative experiments were carried out on the self-constructed M-PDD dataset. As shown in Table 6, we demonstrate that the proposed methodology demonstrates remarkable advantages in all evaluation metrics, further confirming its efficacy.

As shown in Table 7 and Table 8, this study conducted performance comparisons between the baseline model (RT-DETR) and the optimized model (LRD-DETR) on UAV-PDD2023 and M-PDD, respectively. Experimental data confirm that the LRD-DETR model achieves remarkable progress in both precision and recall for distress categories such as cracks, potholes, and repairs. Specifically, this model effectively enhances the detection accuracy for various types of pavement distress while significantly reducing the rate of missed detections. This technical advancement provides a scientifically sound and reliable methodology for highway maintenance, which offers substantial practical value to optimize road preservation efficiency and ensure transportation safety.

**Figure 7:**
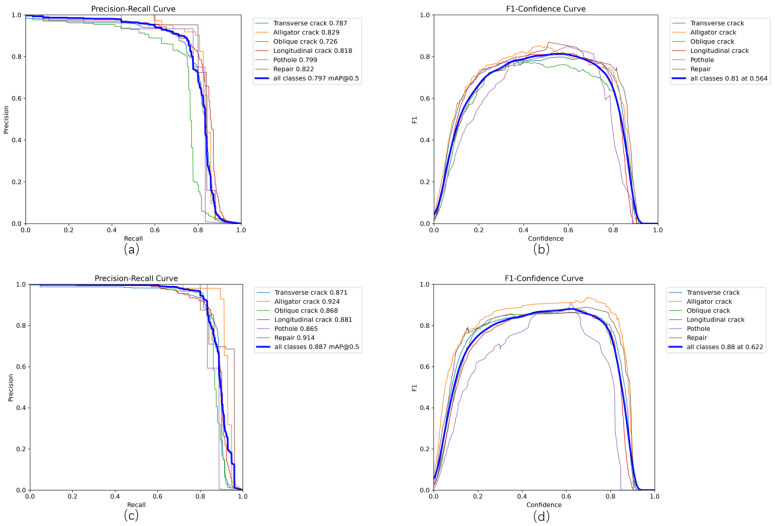
(**a**,**b**) represent the precision–recall curve and F1–confidence curve of the RT-DETR, respectively; and (**c**,**d**) represent the precision–recall curve and F1–confidence curve of the LRD-DETR.

Figure 7 presents the precision–recall (P-R) curves and F1–confidence curves of the baseline model and the proposed improved model on the UAV-PDD2023 dataset. The integral of the precision–recall curve, quantified as the average precision (AP), serves as a comprehensive indicator of model performance, where elevated AP scores correspond to enhanced detection proficiency. The comparative analysis of Figure 7a,c reveals a notable increase in AP for the proposed model. The apex of the F1–confidence curve corresponds to the confidence level that optimizes the F1-score, establishing an ideal trade-off between precision and recall. This threshold plays a critical role in filtering predictions, and higher values indicate more reliable detection outcomes. Figure 7b,d clearly illustrate that the enhanced model attains superior F1-score values and higher optimal confidence thresholds relative to the baseline architecture. The experimental evidence unequivocally demonstrates that LRD-DETR surpasses RT-DETR in precision and recall dimensions, substantiating the efficacy of the architectural modifications introduced.

**Figure 8:**
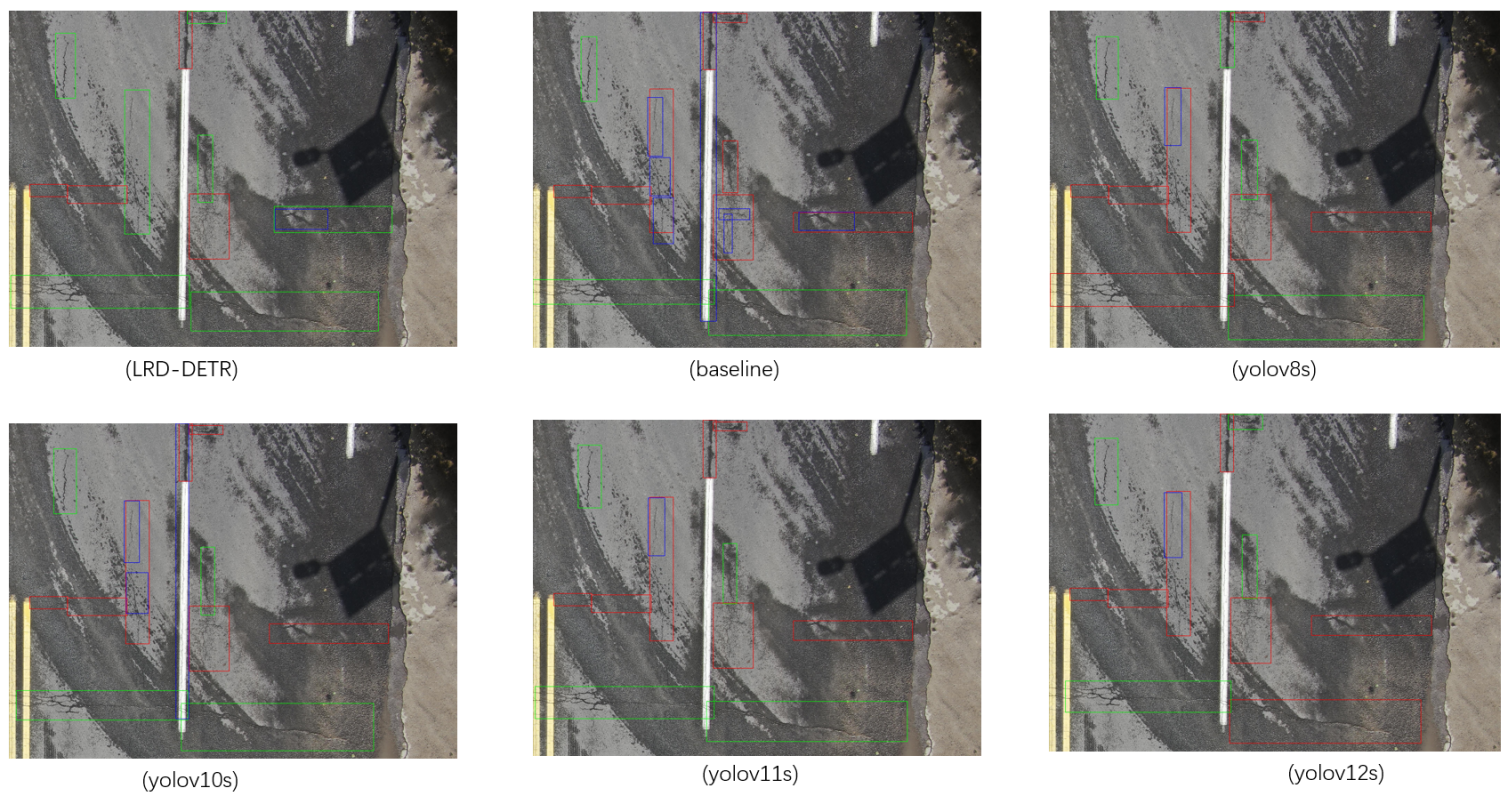
Detection performance comparison among LRD-DETR, RT-DETR, and YOLO series models, where bounding box colors correspond to detection outcomes: green for correct, red for missed, and blue for false detections.

Figure 8 shows the detection results for the RT-DETR, LRD-DETR, and YOLO series models, where the colors of the bounding box correspond to the detection outcomes as follows: green for correct, red for missed, and blue for false detections. Visual evidence from the figure demonstrates that, relative to the RT-DETR framework, the LRD-DETR model exhibits fewer red missed detection boxes and blue false detection boxes in the detection results, while the number of green correct detection boxes increases significantly. This observation confirms that the introduced module can significantly strengthen the feature representation of pavement distress and suppress background interference, improving the model’s recognition robustness and localization accuracy in complex road environments, which further validates the effectiveness of the model architecture design. LRD-DETR was trained and validated on the UAV-PDD2023 and M-PDD datasets, respectively. The specific detection results are shown in Table 7 and Table 8. The experimental outcomes demonstrate that the LRD-DETR model significantly improves both the detection precision and computational performance of pavement defect identification on complex backgrounds.

**Figure 9:**
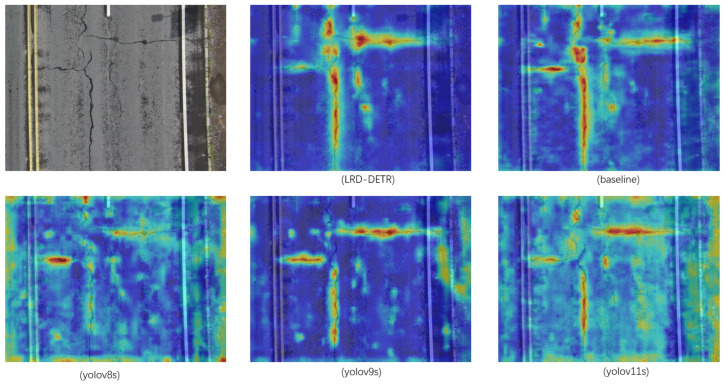
The focusing ability of different detection models on distress features under normal pavement.

**Figure 10:**
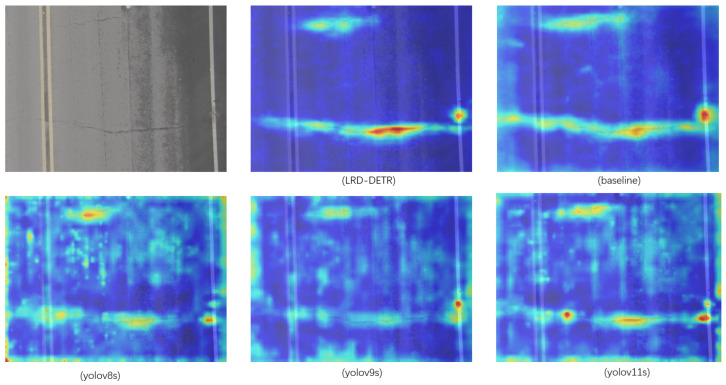
The focusing ability of different detection models on distress features under foggy weather.

Figure 9 and Figure 10 present the visualized detection outcomes of the baseline, LRD-DETR, and selected YOLO-based models under the conditions of normal pavement and foggy weather, respectively. Analysis of the heatmaps (the visualization technology used is Grad-CAM++, because it is used to screen the features of pavement distress, and the confidence threshold is set to 0.4) reveals that the LRD-DETR model exhibits significantly reduced interference from background noise and demonstrates more discriminative response of characteristics to the regions of pavement distress.

## 5. Discussion

In practical pavement distress detection scenarios, the deployment of an unmanned aerial vehicle (UAV) effectively reduces labor costs, mitigates safety risks, and minimizes traffic flow disruption. However, road image collection often faces the problem of dual complications of the background environment and distress characteristics, posing significant challenges for the RT-DETR model in attaining precise and computationally efficient detection.

To overcome these challenges, the present work introduces an LRD-DETR model. Through synergistic integration of feature enhancement, multi-scale feature fusion, and model lightweighting strategies, the proposed model achieves remarkable enhancements in both precision and recall to detect various types of pavement distress, outperforming existing detection models. Specifically, precision and recall rates reached 93.3% and 83.2%, and mAP@0.5 achieved 88.7%. In contrast to the RT-DETR framework, the proposed model improved precision, recall, and mAP@0.5 by 4.9%, 8.7%, and 9.0%, correspondingly, coupled with a 37.9% reduction in parameter volume and a 43.8% decrease in computational overhead. The empirical findings comprehensively substantiate the dual advantages of the developed approach that encompasses detection precision and computational efficiency. Furthermore, through quantitative benchmarking assessments with various existing detection models and visual analysis of heatmaps, it is further validated that the devised method for various road distress features under complex road scenarios (such as illumination variations, shadow occlusion, water stain interference, etc.) significantly outperforms existing methods. At the same time, we conducted comprehensive testing on the M-PDD dataset (which encompasses diverse challenging weather and lighting conditions, including rainy days, foggy conditions, and nighttime street lighting), demonstrating that the proposed method effectively enhances the model’s robustness and reliability in practical application environments.

### 5.1. Main Research Contents

First, this study constructed a lightweight backbone network. The LFEM performs adaptive feature extraction on the feature representations from the input, embedded within the C2f architecture, and combined with efficient ADown down-sampling. It can significantly minimize the number of model parameters and achieve model lightweighting while ensuring detection accuracy. The results of the ablation experiment in Table 3 show that the proposed backbone network reduces the quantity of model parameters by 39.8% relative to the original architecture, confirming the validity of its lightweight structure. Second, to enhance foreground features (e.g., texture details of cracks, potholes, and repairs) while suppressing complex background interferences (e.g., illumination variations, stains, and pavement texture noise), this study integrates a frequency-space attention (FSA) module into feature layer S4 and a polarity-aware linear attention mechanism (Pola) into layer S5. The generated attention weights can enhance feature contrast, boosting the model’s detection capability for small target distresses (e.g., fine cracks and potholes) and further enhancing the accuracy of distress detection. Finally, a cross-scale spatial feature fusion module (CSF^2^M) is constructed to integrate multi-level feature representations, comprehensively enhancing the adaptability and precision of the detection model for various types of pavement distress.

**Figure 11:**
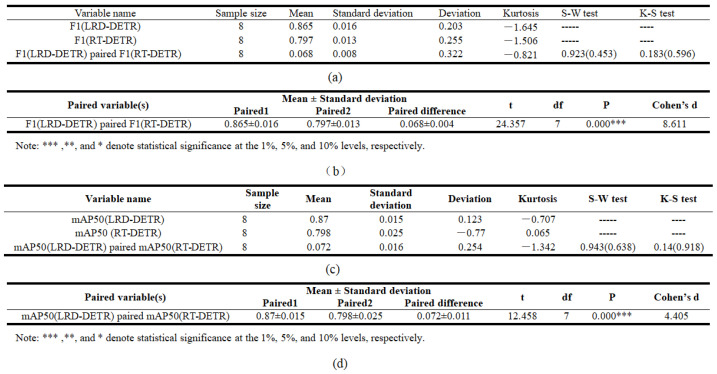
The statistical significance analysis results of LRD-DETR and RD-DETR; (**a**,**c**), respectively, represent the results of the paired difference normality test of F1 and mAP@0.5; (**b**,**d**), respectively, represent the results of the paired sample *t*-test of F1 and mAP@0.5.

To verify that the enhancement in performance of the proposed model originates from its structural improvements rather than random fluctuations, this study conducted eight independent repeated experiments on LRD-DETR and RD-DETR (data shown in Table 9). Representative comprehensive evaluation metrics, namely, the F1 score and mAP@0.5, were selected for paired-sample *t*-tests, respectively. The results of the normality test, as shown in Figure 11a,c, indicate that the samples of paired-differences for both groups follow a normal distribution, satisfying the prerequisite for the *t*-test. The results of the paired *t*-test are presented in Figure 11b,d. In terms of the F1 score, LRD-DETR (0.865 ± 0.016) significantly outperforms RD-DETR (0.797 ± 0.013), with an average improvement of 0.068 (t(7) = 24.357, *p* < 0.001). Similarly, for mAP@0.5, LRD-DETR (0.87 ± 0.015) also shows a significant superiority over RD-DETR (0.798 ± 0.025), with an average improvement of 0.072 (t(7) = 12.485, *p* < 0.001). This improvement exhibits a remarkable effect size (Cohen’s d = 4.405), demonstrating that the improvement in performance is not only statistically significant, but also possesses substantial practical relevance. These results provide strong statistical evidence confirming the efficacy of the proposed module in improving the detection capability of pavement distress.

### 5.2. Limitations and Future Work

The findings presented in Table 3 validate the effectiveness of this model. However, this study still has room for improvement in both detection performance and model lightweighting. Future work could be further developed in the following three areas: First, we expand the multi-scenario pavement distress dataset (covering different weather conditions, pavement materials, and lighting environments), integrating multimodal perception data such as infrared images, LiDAR point clouds, and millimeter-wave radar to enhance model robustness against complex real-world interferences. Second, we incorporate domain adaptation techniques to mitigate domain shift arising from variations in data sources and environmental conditions. This technique has been validated in multiple computer vision tasks as follows: in construction safety monitoring, a Faster R-CNN model based on adversarial domain adaptation at both image and instance levels achieved detection of personal protective equipment (PPE) violations across different viewpoints and imaging conditions [38]; in urban building information extraction, Transformer-based architectures combined with domain-adversarial learning and pseudo-label self-training significantly improve cross-domain generalization for building facade material recognition [39]. These validated domain adaptation methods provide a transferable framework for enhancing cross-domain adaptability in pavement distress detection, and it can effectively reduce the distribution difference between the training dataset (source domain) and real-world road distress (target domain), boosting the model’s generalization capacity and detection accuracy of the model. Third, we further explore model quantization, pruning, and other lightweighting techniques. By optimizing the structure of the model through compression, we improve the efficiency of deployment on computationally constrained platforms, such as edge devices, thereby accelerating the translation of algorithmic results into engineering practice.

## 6. Conclusions

This research develops the LRD-DETR architecture, an optimized pavement distress detection model based on RT-DETR, which effectively addresses the main difficulties that include complicated backgrounds, notable scale disparities, and the excessive parameterization of the model. The key innovations and performance gains are summarized as follows. First, the new backbone network constructed by combining C2f-LFEM and ADown reduced the number of model parameters by 39.8% compared to the original model, verifying the efficacy of its lightweight design. Second, the introduction of Pola and FSA enhanced the expression of disease features of small targets and suppressed the interference of complex backgrounds, resulting in the precision and recall statistics of the approach reaching 91.5% (an increase of 3.1%) and 83.6% (an increase of 9.1%), respectively, and mAP@0.5 increasing to 85.8% (an increase of 6.1%). Finally, the proposed CSF^2^M based on the RCM enhances the model’s emphasis on diverse distress types and improves adaptability to multi-scale defects (e.g., fine cracks vs. extensive cracking). The precision and recall values of the framework reached 93.3% (an increase of 4.9%) and 83.2% (an increase of 8.7%), respectively, and mAP@0.5 reached 88.7% (an increase of 9.0%), achieving a 38.0% decrease in parameter scale and a 43.8% reduction in computational overhead. LRD-DETR achieves significantly enhanced robustness in complex road inspection scenarios through its synergistic feature enhancement module and cross-scale feature fusion mechanism. The inference speed of the framework is 27.8 frames per second on the NVIDIA Jetson Orin Nano (NVIDIA Corporation, Santa Clara, CA, USA) embedded platform, which provides a practical solution for high-precision automatic pavement distress detection and shows great application potential in various maintenance environments, including urban roads, highways, and rural infrastructure.

## Figures and Tables

**Table 1:** Quality assessment of generated pavement distress slices.

Type of Distress	SSIM	FID
Alligator Crack	0.847	18.6
Pothole	0.825	21.3
Repair	0.832	19.8

**Table 2:** Quality assessment of synthetic pavement distress images.

Metric	Balance	Spatial
Score	0.895	0.920

**Table 3:** The influence of the detection results of different improved modules.

Module				Metrics					
Backbone	Pola	FSA (S4)	${\mathrm{CSF}}^{2}M$	P (%)	R (%)	F1 (%)	mAP@0.5	GFLOPs	Param
Baseline				88.4	74.5	80.8	79.7	57.0	19.8
√				87.9	77.4	82.3	83.0	40.5	11.9
	√			90.2	77.7	83.4	84.2	57.2	20.0
		√		92.1	76.8	83.7	83.2	57.0	20.7
			√	91.3	75.1	82.4	84.2	48.2	19.2
√	√			89.1	80.0	84.3	84.8	40.8	12.1
√	√	√		91.5	83.6	87.4	85.8	40.8	12.9
√	√		√	92.6	78.4	84.9	84.6	34.6	12.4
√	√	√	√	93.3	83.2	87.9	88.7	32.0	12.3

backbone = C2f_LFEM+ADown.

**Table 4:** The model detection results of using the FSA module in the S3 and S4 feature layers.

	Metrics						
Model	P (%)	R (%)	F1 (%)	mAP@0.5	FPS	GFLOPs	Param
Baseline	89.1	80.0	84.3	84.8	62	40.8	12.1
+FSA (S3)	88.9	78.6	83.4	84.2	58	40.8	13.7
+FSA (S4)	91.5	83.6	87.4	85.8	57	40.8	12.9

**Table 5:** Compare the detection results of different models–UAV-PDD2023.

	Metrics					
Model	P (%)	R (%)	F1 (%)	mAP@0.5	GFLOPs	Param
Baseline (RT-DETR)	88.4	74.5	80.8	79.7	57.0	19.8
YOLOv5m	84.2	79.8	81.7	84.5	64.0	25.0
YOLOv8m	87.9	64.7	74.5	76.4	78.7	25.8
YOLOv9s	85.9	63.4	72.6	71.5	26.7	7.2
YOLOv10s	77.4	68.4	71.9	76.5	24.5	8.0
YOLOv11m	81.8	79.1	80.2	83.6	67.7	20.0
YOLOv12s	86.7	70.1	76.7	78.8	21.2	9.2
Deformable-DETR	84.8	73.7	78.9	77.6	165.0	38.3
Dino-DETR	86.4	75.2	80.4	79.7	235.0	46.6
Pavement-DETR	89.3	83.8	86.5	87.1	67.5	-
Ours	93.3	83.2	87.9	88.7	32.0	12.3

**Table 6:** Compare the detection results of different models–M-PDD.

	Metrics					
Model	P (%)	R (%)	F1 (%)	mAP@0.5	GFLOPs	Param
Baseline (RT-DETR)	90.8	80.3	85.2	86.6	57.0	19.8
YOLOv8s	92.7	81.4	86.7	87.2	28.4	11.1
YOLOv9s	91.7	80.5	85.7	87.3	26.7	7.2
YOLOv10s	92.0	78.3	84.5	86.3	24.5	8.0
YOLOv11s	92.7	82.0	86.9	86.5	21.3	9.4
YOLOv12s	90.4	81.1	85.5	86.9	21.2	9.2
Ours	94.7	83.0	88.4	88.5	34.6	12.3

**Table 7:** Comparison of the experimental results of RT-DETR and LRD-DETR on the UAV-PDD2023 dataset.

	P (%)					
Model	TC	AC	OC	LC	Pothole	Repair
Baseline	86.5	91.3	85.3	86.2	89.1	92.0
Ours	90.9	91.0	95.7	90.6	94.4	97.1
	R (%)					
Model	TC	AC	OC	LC	Pothole	Repair
Baseline	74.0	74.4	68.4	76.3	77.8	76.0
Ours	81.8	91.2	80.9	81.8	83.3	80.0

**Table 8:** Comparison of the experimental results of RT-DETR and LRD-DETR on the M-PDD dataset.

	P (%)					
Model	TC	AC	OC	LC	Pothole	Repair
Baseline	90.0	96.5	90.9	93.1	97.3	94.4
Ours	91.3	96.6	92.0	95.2	98.4	95.1
	R (%)					
Model	TC	AC	OC	LC	Pothole	Repair
Baseline	77.0	82.1	72.0	77.8	84.7	85.9
Ours	80.0	88.6	77.4	80.5	86.1	87.3

**Table 9:** F1 and mAP@0.5 of RT-DETR and LRD-DETR on the UAV-PDD2023 dataset.

Exp	LRD-DETR (F1)	LRD-DETR (mAP@0.5)	RT-DETR (F1)	RT-DETR (mAP@0.5)
1	0.879	0.887	0.808	0.797
2	0.852	0.848	0.785	0.782
3	0.882	0.892	0.801	0.816
4	0.851	0.862	0.792	0.783
5	0.864	0.876	0.812	0.822
6	0.843	0.859	0.782	0.785
7	0.859	0.865	0.798	0.805
8	0.878	0.873	0.816	0.821

## Data Availability

Some or all the data, models, or codes that support the findings of this study are available from the corresponding author upon reasonable request.
